# Application of Functional Genomics and Proteomics to Plant Cryopreservation

**DOI:** 10.2174/138920210790217945

**Published:** 2010-03

**Authors:** Gayle M Volk

**Affiliations:** USDA-ARS-National Center for Genetic Resources Preservation, 1111 S. Mason St., Ft. Collins, CO 80521, USA

**Keywords:** Cryobiology, cold hardiness, desiccation tolerance, stress, microarray, vitrification.

## Abstract

Plant cryobiology has primarily emerged from the classical fields of cryobiology and plant stress physiology. Cryopreservation tools are now available to geneticists for germplasm preservation and the field itself is advancing significantly through the use of molecular techniques. Long-term preservation of vegetatively propagated tissues can minimize the risks of long-term maintenance under tissue culture or field conditions. Cells can be successfully cryopreserved when the adverse affects of ice crystal formation are mitigated by the removal of water or procedures to limit ice formation and crystal growth. The addition of cryoprotectant solutions to hydrated cells may improve the survival of microdissected shoot tips or embryonic axes. Recent discoveries in the genetic pathways leading to cold acclimation and freezing tolerance suggest the involvement of key cold-regulated genes in the acquisition of cold tolerance in plant tissues. Model systems of banana and *Arabidopsis *have revealed the involvement of genes and proteins in the glycolytic and other metabolic pathways, particularly processes involved in dehydration tolerance, osmoprotection, and membrane transport. Furthermore, successful recovery appears to be dependent upon the presence of antioxidant protection from reactive oxygen species. Characterization of specific genes and proteins will lead to significant advances in plant cryobiology research.

## INTRODUCTION

The field of plant cryobiology seeks to understand the physiological and molecular processes that allow plants to survive low temperatures. The focus of cryobiology is predominantly cryopreservation: the process and methods that permit long term survival of organisms at liquid nitrogen temperatures.

Once cryopreserved, materials remain safe and available for a low annual cost. Novel, mutant, or transformed cell lines that can no longer be maintained in laboratory settings can be cryopreserved to prevent their loss [1]. Clonal lines can be kept as base collections for expensive field plantings [2]. Large quantities of seeds or pollen can also be stored cryogenically as a reference collection for the available genetic diversity of a population or as a source for new alleles in the future [3].

Successful cryogenic storage is dependent upon having a reliable source of coolant as well as a good documentation system. Once achieved, propagules often have extended storage longevity compared to conventional storage regimes (refrigerator or freezer conditions). Genetic drift and mutations are minimized compared to when materials are maintained in an actively growing state for extended intervals [3].

Long term storage reduces the frequency of regenerations of accessions in genebanks and facilitates the use of specific individuals in breeding programs. Availability and transportability of cryopreserved pollen allows breeders to have access to materials that might otherwise be unavailable. Cryopreservation of embryo cultures lets breeding programs prolong the availability of juvenile materials while waiting for characterization of mature tree selections [4]. Cryotherapy has also been used to remove viruses from infected plants [5]. But, cryogenic storage does have its drawbacks: phenotypic characterization is challenging and it may take many years for a recovered propagule to reach reproductive maturity for use in a breeding program.

## GENOMIC OPPORTUNITIES

Plant cryobiology is a research discipline that is beginning to enter into the genomics arena. Traditionally, the discipline has been considered an extension of the cold hardiness and drought tolerance fields, centered primarily on long term preservation of seeds, shoot tips, dormant buds, pollen, embryos, cell lines, and other propagule types. A greater application of genomics techniques can increase our understanding of plant cryobiology. By combining physiological and genomic approaches, an array of methods are available to understand how cells are protected from freezing stress.

One of the key issues in plant cryobiology is understanding why some genotypes are less tolerant of preservation stresses than other genotypes. Widescale adoption of cryopreservation technologies will likely depend on identifying universal cryoprotection protocols for each propagule type. A characterization of the genetic response to diverse cryoprotectants will determine if methods that rely on rapid cellular desiccation, permeable cryoprotectants, freeze desiccation, or air desiccation all result in similar cellular responses. Do cells sense and respond to the stresses differentially? Alternatively, do cells ultimately respond to either desiccation and/or temperature extremes? Careful analyses of gene expression responses will reveal which stress is perceived by cellular machinery and the pathways that are activated.

Seeds and dormant buds can acquire an endogenous tolerance to desiccation and cryogenic temperature stresses. Dormant buds from temperate species collected mid-winter are more amenable to cryopreservation than those collected prior to acquiring cold tolerance or those which lose their cold-hardiness in the springtime [6]. This acclimation process is dependent upon maturation, climatic conditions, daylength, and genotype. Temperate species in tissue culture can often be acclimated to cold and improve cryopreservation success [7, 8]. It is presumed that an understanding of the acclimation process will provide insights into how propagules can be exogenously treated to tolerate cryogenic stresses.

The process of propagule recovery is poorly understood. Do cells acquire adequate protection from undesirable chemical effects (such as free radicals) that are generated during recovery? Is it possible to minimize production of free radicals while promoting cellular repair? Comparison of gene expression patterns will provide key information as to the timing and nature of the recovery pathways in systems that survive or fail to survive cryopreservation.

Molecular marker technologies have already been applied to cryopreserved plant materials to ascertain if recovered explants are true-to-type. Generally, there is consensus that plant genotypes remain stable after cryopreservation when a single genotype is the conservation target [9-13]. Key discoveries have revealed that DNA methylation patterns may be affected by cryoprotectant and/or liquid nitrogen exposure [14, 15]. Variation in DNA methylation patterns may cause the apparent genomic modifications when methylation sensitive marker systems are employed [16]. In addition, phenotypic variation may result from other procedures, such as tissue culture, that are often used in cryopreservation methods [17].

## COLD AND DESICCATION STRESS

Both dehydration and low temperature affect water relations within the cell. Lethal ice forms when hydrated cells are cooled below their freezing point. Thus, for successful cryopreservation, cells must be desiccated to prevent ice propagation. Cells must also have intact membranes and stable proteins to tolerate the desiccation and cold stresses. The rich literatures of cold and desiccation tolerance have revealed that extraordinarily similar response pathways are triggered by these different stresses.

It is not surprising that the regulatory mechanisms for desiccation and cold tolerance partially overlap. These stresses invoke second messengers such as calcium, reactive oxygen species, and inositol phosphates within minutes of stress signal perception [18]. The cascades resulting from these signal molecules result in the expression of transcription factors that affect the gene expression of hundreds of stress response genes.

Microarray chip methods have identified 299 drought inducible, 54 cold inducible, and 245 abscisic acid (ABA) inducible genes in *Arabidopsis* [19, 20]. Of the drought inducible genes, more than half were also induced by ABA, but only 105 of the drought inducible genes were induced by cold. Classes of drought inducible genes included: chaperones, LEA (Late Embryogenesis Abundant), osmotin, antifreeze, mRNA binding, osmolyte biosynthesis, water channel proteins, sugar and proline transporters, proteases, and detoxification enzymes. Recently, small RNAs specific to abiotic stresses have also been identified [21]. In this review, gene expression patterns in response to desiccation and cold stress literature will be briefly summarized since those subjects warrant reviews of their own.

### Cold Stress

Cold treatments cause abrupt changes in the structure of cellular lipids and cytoplasm. Ice forms in hydrated cells and the plasma membrane undergoes an undesirable phase change that is induced by low temperatures. Formation of extracellular ice removes freezable cellular water, but also invokes a desiccation stress.

Cold acclimation treatments can increase the levels of unsaturated fatty acids, alter lipid and protein composition, change the lipid/protein ratio, and increase the proportion of phospholipids to stabilize membranes and prevent phase changes [22, 23]. Lipoproteins and ERD14 (Early Response to Dehydration protein 14) increase during cold acclimation and are believed to encourage the formation of exocytotic extrusions instead of endocytotic vesicles during freeze induced osmotic contraction [22, 24, 25]. This process is facilitated by a change in gene expression patterns for the responsible proteins. Other cold-regulated proteins, such as cryoprotectin, protect membranes from freeze-thaw damage [26]. Cells also respond to cold acclimation treatments by producing proteins that affect the accumulation of osmolytes such as proline, betaine, polyols, and sugars [27]. These osmolytes may decrease the cellular freezing temperature through freezing point depression, but this does not provide adequate protection from cryogenic temperatures. However, some sugars, such as sucrose, may also interact with membranes and other proteins (such as tocopherol) to improve membrane stability during freezing [28].

Genomic evaluations of cold acclimated and exposed plants have revealed complex response pathways. The DREB1 proteins are transcriptional activators of over 300 genes in the CBF cold response pathway [29]. Transcription factors CBF1, CBF2, and CBF3 (or DREB1b, DREB1c, and DREB1a) are induced within an hour after exposure to 4^o^C [29]. The cold stress response is mediated by these transcription factors interacting with the cis-acting elements DRE/CRT regulatory elements in promoters of key genes [20]. The CBF/DREB transcription factors also affects expression of genes such as COR15a (protection of proteins against freeze-inactivation), RD29a, and those that encode LEA proteins [29].

### Desiccation Stress

Desiccation tolerant seeds and pollen undergo extreme dehydration as part of the developmental maturation process. Orthodox seeds can lose most of their moisture during maturation, resulting in moisture contents of around 8%. At low moisture content, the cytoplasm enters a glassy state where molecular movement is minimal [30]. Glasses maintain the structural and functional integrity of macromolecules and prevent membrane fusion to limit the extreme deformation imposed by desiccation [31].

Vegetative cells can also tolerate desiccation. Tonoplast and plasma membrane specific aquaporins, water channels across membranes, may play critical roles in water movement during both desiccation and rehydration. Desiccation stresses may cause the plasma membrane to lose surface area. Sugars and other polyhydric solutes are believed to protect protein and membrane structures during desiccation, though the mode of action remains poorly understood [31, 32].

LEA proteins accumulate in seeds when desiccation tolerance is acquired during seed maturation. LEA proteins are hydrophilic and remain soluble at boiling temperatures (e.g. [33]). They may also have protein and membrane stabilization properties [34]. Dehydrins are a class of LEA proteins that are induced by ABA and are suggested to inhibit the denaturation of macromolecules (e.g. [35]).

#### A. ABA Independent Pathways

The genetic response to drought or desiccation has been identified in several model systems. ABA independent pathways ultimately affect the pathways that regulate osmotic equilibrium and detoxification [36]. 

The DRE/CRT cis-acting element associates with the desiccation-specific DREB2 transcription factor, found in the ABA independent pathway response to drought stress [37, 38]. ERD1 is another key gene involved in the ABA independent stress-response. The NAC group of transcription factors binds to the elements within the ERD1 gene promoter [39, 40]. Another NAC transcription factor, RD26, is dehydration responsive. RD26-regulated genes may play a role in the detoxification of reactive oxygen species [41].

#### B. ABA Dependent Pathways

Exogenous ABA induces genes that are also activated by dehydration. In *Arabidopsis*, several hundred genes were induced by exogenous ABA treatment [42]. An exogenous ABA treatment also improves cryopreservation success for some, but not all, species [43-46]. 

Endogenous ABA is essential for some drought stress-responses [47], including the production of LEA proteins [48]. LEA proteins are thought to improve the macromolecular stability of seeds due to their stronger hydrogen bonding strength [48]. Most LEA proteins have either low temperature response elements or ABRE in their promoters [49]. The ABF/AREB transcription factors are basic leucine zipper types that bind to the ABRE elements and activate stress gene expression. Genes AREB1 and AREB2 are ABA responsive [18].

MAP kinases also play a role in the regulation of stress responses by affecting protein phosphorylation and dephosphorylation states [41]. MAP kinases can be induced by cold, salt, drought, ABA and reactive oxygen species. The MAPK cascades improve reactive oxygen species scavenging capacity.

An alternative ABA dependent pathway involves the MYC and MYB transcription factors that bind to MYB and MYC recognition sequences and activate the drought inducible RD22 gene [18].

### Recovery

Survival after desiccation is dependent upon cellular protection from the stress of water loss as well as the availability of functional repair mechanisms after rehydration. Desiccation tolerant seeds and pollen generally survive rehydration; whereas it is much more challenging to successfully regenerate less tolerant propagule types. Recovery is dependent upon having enough cells survive for successful propagule regeneration. In shoot tip systems, many of the larger cells in regions surrounding the meristem become extremely plasmolyzed and do not survive cryopreservation [50].

The presence of reactive oxygen species within cells after stress can damage cells and generate lipid peroxides, aldehydic products and protein carbonyls [48, 51]. High antioxidant status is associated with tolerance to cryopreservation. Cryo-tolerant *Ribes* accessions exhibited higher hydroxyl radical activity, antioxidant status, phenolic accumulation and anthocyanin pigments than *Ribes* accessions that were cryo-sensitive [52]. The reactive oxygen species gene network includes over 150 genes [53]. Reactive oxygen species can be scavenged by enzymes that are induced by both cold and drought stresses. Heat shock proteins can also be activated by reactive oxygen species [54]. The cytoprotective properties of heat shock proteins help maintain proteins in functional conformations and prevent aggregation [54].

## EXPERIMENTAL SYSTEMS

**Fig. (1):**
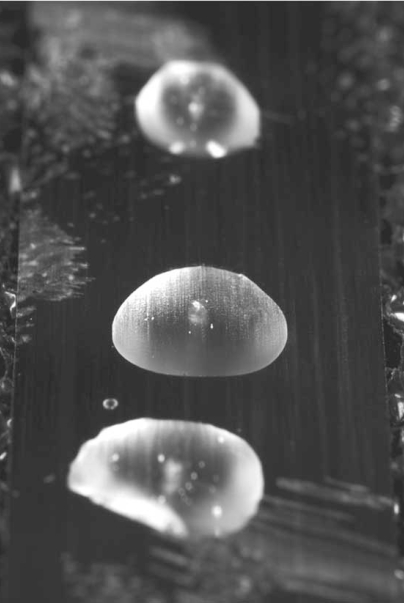
Plant shoot tips (1 mm diameter) were immersed in droplets of PVS2 cryoprotectant solution on a foil strip, cooled to -196°C and photographed. Transparent nature of droplet suggests that ice did not form during the cooling process. Photo courtesy of Stephen Ausmus, USDA-ARS.

Many plant cryobiologists have focused on finding methods to conserve horticultural species that are vegetatively propagated since seed storage does not maintain the genotype of interest. Recently, a cryopreservation protocol has been published for *Arabidopsis* T87 suspension cells to facilitate conservation of transgenic lines. *Arabidopsis* can also be a good model to identify the gene expression responses to cryogenic stress [55]. *Arabidopsis* shoot tips are easy to produce and readily survive diverse cryo-exposure protocols [56]. In 2006, Towill *et al*. published methods for the cryopreservation of *Arabidopsis* shoot tips using three of the more common shoot tip cryoprotection protocols. Cryoprotectants serve to both dehydrate and promote the glassy state within cells [57]. Published *Arabidopsis* methods employed the cryoprotectants Plant Vitrification Solution 2 (PVS2; 30% glycerol, 15% ethylene glycol, 15% DMSO, 15% sucrose; [58]), Plant Vitrification Solution 3 (PVS3; 50% sucrose, 50% glycerol, [59]) and PGD (10% polyethylene glycol, 10% glucose, 10% dimethyl sulfoxide; [7]). Once treated with vitrification-type cryoprotectants PVS2 and PVS3, shoot tips can often be rapidly cooled to LN temperatures and stored for extended lengths of time. Shoot tips are either cooled within cryoprotectant droplets (Fig. **1**) or in solution-filled cryovials. In contrast, PGD-treated shoot tips are slowly cooled within cryovials to -30 or -35^o^C prior to LN exposure.

Cryoprotectants vary in their toxicity as well as their protective mechanisms [60, 61], thus providing an experimental system by which the conservation of stress response pathways across methods can be compared. Basu [62] identified some candidate genes, such as a calcium ion binding protein, that were upregulated in response to PVS3 exposure using the *Arabidopsis* system. Comparisons of gene expression patterns during cryoprotectant treatment, liquid nitrogen exposure, and recovery of *Arabidopsis* shoot tips using cDNA microarrays have revealed suites of genes up- and down-regulated in shoot tips that survive cryopreservation (Volk *et al*. in prep.).

*Musa* shoot tips from multiple species are amenable to vitrification methods [63, 64]. Since little genomic information is available for *Musa,* proteomics approaches have proved advantageous [65-67]. Meristem-specific proteins expressed in response to high sucrose pretreatments include those involved in glycolysis and maintaining cell wall integrity [68]. A website has been established for *Musa* proteomics efforts (http://www.pdata.ua.ac.be/musa/ modules/listview/?table=spot). These high sucrose treatments serve to decrease *Musa* meristem water content and increase the intracellular sucrose concentration [69].

Progress in genomic research in other kingdoms may reveal fundamental stress responses of cells to extreme conditions. Insects and microbes have adapted to polar environments [70]. Reptiles and amphibians survive harsh Canadian winters with distinct, yet similar responses.

Desiccation and cold response pathways are interrelated in insects. Cold acclimated microarthopods (*Cryoptopygus antarcticus*) from Antarctic regions upregulate structural and cuticle proteins. Furthermore, expression of genes involved in moulting supports a role of moulting in cold tolerance [71]. Arctic springtails (*Onychiurus arcticus*) have also served as model to study desiccation and cold tolerance in insects [72]. As in plants, springtails increase the proportion of unsaturated to saturated fatty acids in lipid membranes. The sugar trehalose also serves to stabilize membranes presumably by interacting with phospholipids in a water-replacement like mechanism [72]. Sequenced EST libraries from *O. arcticus* in various states of desiccation identified clones representing genes that fall into classes of aquaporins, dehydrins, heat shock proteins and those relating to antioxidant production (glutathione, catalase, hydrogen peroxidase) that were upregulated [73]. Subtractive techniques revealed recovery genes such as heat shock proteins, membrane proteins, and those involved in metabolic pathways [72].

The Storey laboratory at Carleton University has made remarkable progress identifying genomic responses in cold-tolerant vertebrates. Frogs and reptiles overwinter in a frozen state and use ice nucleators to initiate the freezing process extracellularly and use antifreeze proteins to inhibit recrystallization during freezing. High osmolyte contents limit cell volume reduction, membrane stabilizers such as trehalose and proline prevent lipid biolayer compression, and physiological adaptations regulate the cessation and reactivation of breathing and the heart [74]. Novel genes with low copy number transcripts were identified using microarrays and tracked responses by groups of genes [75]. Freeze tolerance and anoxia exposure microarrays both revealed genes involved in iron binding, antioxidant defense, and serine protease inhibitors [76]. Genes that affect the production of low molecular weight osmolytes and provide antifreeze protection are upregulated [76]. In addition, protection mechanisms such as heat shock proteins, glutathione peroxidase, and glutathione S transferase, and peroxiredoxin are activated [76].

Successful cryoprotection in both animals and plants is dependent upon minimizing freezeable water within cells and the maintenance of sufficient cell volumes [74, 77]. Cold tolerant living systems can often sequester excess water extracellularly to limit ice formation in the cytoplasm [77]. Cold tolerant animals and plants both exhibit forms of cold acclimation, with protective changes occurring internally on a seasonal basis [6, 76]. Freeze avoiding insects make glycerol as an internal cryoprotectant [76, 77]; whereas in plants, glycerol is frequently added in the form of cryoprotectant solutions to help protect cells during cryopreservation. Membrane fluidity is key to survival of cold conditions. Short chain and unsaturated fatty acids maintain membrane fluid states promote chilling tolerance across diverse species [22, 23, 77]. Furthermore, either high antioxidant levels or an efficient method of removing reactive oxygen species are key to successful recovery after cryoexposure in both the plant and animal kingdoms [52, 76, 77].

It is clear that similar mechanisms have evolved in diverse kingdoms that enable organisms to tolerate extreme temperatures and desiccation. Cells have reduced biochemical reaction rates, increased cellular viscosities, alterations in membrane lipids and changes in protein conformation in response to extremely cold conditions [77]. By minimizing ice nucleation and promoting glassy states, cells can survive extreme stresses. Their survival is dependent upon producing proteins and chaperones that will protect against potentially dangerous reactive oxygen species. 

## CONCLUSIONS 

Prevention of cellular ice formation and maintenance of intact membranes are critical for successful plant cryopreservation. Identification of the genomic responses to cold and desiccation stresses during the cryopreservation process will reveal how propagules respond to diverse cryoprotectants, extreme temperatures, and recover. Understanding plant acclimation is key to determining endogenous responses and more information about the recovery process is needed. Comparing the genetic responses to tolerance and longevity within and among plant species and propagules will guide conservation scientists in their quest to design improved preservation strategies.

## References

[1] Ericsson C, Franzen B, Nister M (2006). Frozen tissue biobanks. Tissue handling, cryopreservation, extraction, and use for proteomic analysis. Acta Oncologica.

[2] Tsai C.-J, Hubscher S.L. (2004). Cryopreservation of *Populus* functional genomics. New Phytol.

[3] Walters C, Volk G.M., Richards C.M. (2008). Genebanks in the post-genomic age: Emerging roles and anticipated uses. Biodiversity.

[4] Hargreaves C, Maas S, Reeves C, Holden G, Menzies M, Kumar S, Foggo M (2004). Cryopreservation of *Pinus radiata* zygotic embryo cotyledons: effect of storage duration on adventitious shoot formation and plant growth after two years in the field. Can. J. Forest Res.

[5] Engelmann F (2004). Plant cryopreservation: Progress and prospects. In Vitro Cell. Dev. Biol.-Plant.

[6] Tyler N, Stushnoff C (1988). Dehydration of dormant apple buds at different stages of cold-acclimation to induce cryopreservability in different cultivars. Can. J. Plant Sci.

[7] Chang Y.J., Reed B.M. (1999). Extended cold acclimation and recovery medium alteration improve regrowth of *Rubus* shoot tips following cryopreservation. CryoLetters.

[8] Senula A, Keller E.R.J., Sanduijav T, Yahannes T (2007). Cryopreservation of cold-acclimated mint (*Mentha* spp.) shoot tips using a simple vitrification protocol. CryoLetters.

[9] Harding K, Benson E.E. (2000). Analysis of nuclear and chloroplast DNA in plants regenerated from cryopreserved shoot-tips of potato. CryoLetters.

[10] Harding K, Benson E.E. (2001). The use of microsatellite analysis in *Solanum tuberosum* L. *in vitro* plantlets derived from cryopreserved germplasm. CryoLetters.

[11] Kaity A, Ashmore S.E., Drew R.A., Dulloo M.E. (2008). Assessment of genetic and epigenetic changes following cryopreservation in papaya. Plant Cell Rep.

[12] Liu Y.-G., Liu L.-X., Wang L, Gao A.-Y. (2008). Determination of genetic stability in surviving apple shoots following cryopreservation by vitrification. CryoLetters.

[13] Turner S, Krauss SL, Bunn E, Senaratna T, Dixon K, Tan B, Touchell D (2001). Genetic fidelity and viability of *Anigozanthos viridis* following tissue culture, cold storage and cryopreservation. Plant Sci.

[14] Johnston J.W., Benson E.E., Harding K (2009). Cryopreservation induces temporal DNA methylation epigenetic changes and differential transcriptional activity in *Ribes* germplasm. Plant Physiol. Biochem.

[15] Peredo E.L., Arroyo-Garcia R, Reed B.M., Revilla M.A. (2008). Genetic and epigenetic stability of cryopreserved and cold-stored hops (*Humulus lupulus* L.). Cryobiology.

[16] Jaligot E, Rival A, Beule T, Dussert S, Verdeil J.L. (2000). Somaclonal variation in oil palm (*Elaeis guineensis* Jacq.): the DNA methylation hypothesis. Plant Cell Rep.

[17] Harding K, Staines H (2001). Biometric analysis of phenotypic characters of potato shoot-tips recovered from tissue culture, dimethyl sulphoxide treatment and cryopreservation. CryoLetters.

[18] Mahajan S, Tuteja N (2005). Cold, salinity and drought stresses: An overview. Arch. Biochem. Biophys.

[19] Seki M, Narusaka M, Ishida J, Nanjo T, Fujita M, Oono Y, Kamiya A, Nakajima M, Enju A, Sakurai T, Satou M, Akiyama K, Taji T, Yamaguchi-Shinozaki K, Carninici P, Kawai J, Hayashizaki Y, Shinozaki K (2002). Monitoring the expression profiles of 7000 *Arabidopsis* genes under drought, cold, and high-salinity stresses using a full-length cDNA microarray. Plant J.

[20] Shinozaki K, Yamaguchi-Shinozaki K (2007). Gene networks involved in drought stress response and tolerance. J. Exp. Bot.

[21] Sunkar R, Chinnusamy V, Zhu J, Zhu J.-K. (2007). Small RNAs as big players in plant abiotic stress responses and nutrient deprivation. TRENDS Plant Sci.

[22] Uemura M, Tominaga Y, Nakagawara C, Shigematsu S, Minami A, Kawamura Y (2006). Responses of the plasma membrane to low temperatures. Physiologia Plant.

[23] Xin Z, Browse J (2000). Cold comfort farm: The acclimation of plants to freezing temperatures. Plant Cell Environ.

[24] Gordon-Kamm W.J., Steponkus P.L. (1984). The behavior of the plasma membrane following osmotic contraction of isolated protoplasts: implications in freezing injury. Protoplasma.

[25] Gordon-Kamm W.J., Steponkus P.L. (1984). Lamellar-to-hexagonal II phase transitions in the plasma membrane of isolated protoplasts after freeze-induced dehydration. Proc. Natl. Acad. Sci.

[26] Hincha D.K. (2002). Cryoprotectin: a plant lipid-transfer protein homologue that stabilizes membranes during freezing. Phil. Trans. R. Soc. Lond. B.

[27] Levitt J (1980). Responses of plants to environmental stresses.

[28] Hincha D.K. (2008). Effects of β-tocopherol (vitamin E) on the stability and lipid dynamics of model membranes mimicking the lipid composition of plant chloroplast membranes. FEBS Lett.

[29] Fowler S, Thomashow M.F. (2002). Arabidopsis transcriptome profiling indicates that multiple regulatory pathways are activated during cold acclimation in addition to the CBF cold response pathway. Plant Cell.

[30] Vertucci C.W., Farrant J.M., Kigel J, Galili G (1995). Acquisition and loss of desiccation tolerance, in Seed Development and Germination.

[31] Walters C, Koster K.L., Jenks M.A., Wood A (2007). Structural dynamics and desiccation damage in plant reproductive organs, in Plant Desiccation Tolerance.

[32] Uemura M, Steponkus P.L. (2003). Modification of the intracellular sugar content alters the incidence of freeze-induced membrane lesions of protoplasts isolated from *Arabidopsis thaliana* leaves. Plant Cell Environ.

[33] Alpert P, Oliver M.J., Black M, Pritchard H.W. (2002). *Drying without dying*, in 2002. Desiccation and survival in plants: Drying without Dying.

[34] Tunnacliffe A, Wise M.J. (2007). The continuing conundrum of the LEA proteins. Naturwissenschaften.

[35] Pammenter N.W., Berjak P (1999). A review of recalcitrant seed physiology in relation to desiccation-tolerance mechanisms. Seed Sci. Res.

[36] Tran L.-S. P., Nakashima K, Shinozaki K, Yamaguchi-Shinozaki K (2007). Plant gene networks in osmotic stress response: From genes to regulatory networks. Methods Enzymol.

[37] Liu Q, Kasuga M, Sakuma Y, Abe H, Miura S, Yamaguchi-Shinozaki K, Shinozaki K (1998). Two transcription factors, DREB1 and DREB2, with an EREBP/AP2 DNA binding domain separate two cellular signal transduction pathways in drought- and low-temperature-responsive gene expression, respectively, in Arabidopsis. Plant Cell.

[38] Nakashima K, Ito Y, Yamaguchi-Shinozaki K (2009). Transcriptional regulatory networks in response to abiotic stresses in *Arabidopsis* and grasses. Plant Physiol.

[39] Agarwal P.K., Agarwal P, Reddy M.K., Sopory S.K. (2006). Role of DREB transcription factors in abiotic and biotic stress tolerance in plants. Plant Cell Rep.

[40] Tran L.S.P., Nakashima K, Sakuma Y, Simpson S.D., Fujita Y, Maruyama K, Fujita M, Seki M, Sinhozaki K, Yamaguchi-Shinozaki K (2004). Isolation and functional analysis of *Arabidopsis* stress-inducible NAC transcription factors that bind to a drought-responsive cis-element in the early responsive to dehydration stress 1 promoter. Plant Cell.

[41] Fujita M, Fujita Y, Noutoshi Y, Takahashi F, Narusaka Y, Yamaguchi-Shinozaki K, Shinozaki K (2006). Crosstalk between abiotic and biotic stress responses: a current view from the points of convergence in the stress signaling networks. Curr. Opin. Plant Biol.

[42] Yamaguchi-Shinozaki K, Shinozaki K (2006). Transcriptional regulatory networks in cellular responses and tolerance to dehydration and cold stresses. Annu. Rev. Plant Biol.

[43] Burritt D.J. (2008). Efficient cryopreservation of adventitious shoots of *Begonia x erythrophylla* using encapsulation-dehydartion requires pretreatment with both ABA and proline. Plant Cell Tiss. Organ Cult.

[44] Chang Y, Reed B.M. (2001). Preculture conditions influence cold hardiness and regrowth of *Pyrus cordata* shoot tips after cryopreservation. HortScience.

[45] Kendall E.J., Kartha K.K., Qureshi J.A., Chermak P (1993). Cryopreservation of immature spring wheat zygotic embryos using an abscisic acid pretreatment. Plant Cell Rep.

[46] Vandenbussche B, Leuridan S, Verdoodt V, Gysemberg M, De Proft M (1999). Changes in sugar content and fatty acid composition of *in vitro* sugar beet shoots after cold acclimation: imfluence on survival after cryopreservation. Plant Growth Regulation.

[47] Seki M, Umezawa T, Urano K, Shinozaki K (2007). Regulatory metabolic networks in drought stress responses. Current Opin. Plant Biol.

[48] Oliver A.E., Leprince O, Wolkers W.F., Hincha D.K., Heyer A.G., Crowe J.H. (2001). Non-disaccharide-based mechanisms of protection during drying. Cryobiology.

[49] Hundertmark M, Hincha D.K. (2008). LEA (Late Embryogenesis Abundant) proteins and their encoding genes in *Arabidopsis thaliana*. BMC Genomics.

[50] Volk G.M., Caspersen A.M. (2007). Plasmolysis and recovery of different cell types in cryoprotected shoot tips of *Mentha X piperita*. Protoplasma.

[51] Blagojevi? D.P. (2007). Antioxidant systems in supporting environmental and programmed adaptations to low temperatures. CryoLetters.

[52] Johnston J.W., Harding K, Benson E.E. (2007). Antioxidant status and genotypic tolerance of *Ribes in vitro* cultures to cryopreservation. Plant Sci.

[53] Suzuki N, Mittler R (2006). Reactive oxygen species and temperature stresses: A delicate balance between signaling and destruction. Physiol. Plant.

[54] Timperio A.M., Egidi M.G., Zolla L (2008). Proteomics applied on plant abiotic stresses: Role of heat shock proteins (HSP). J. Proteomics.

[55] Ogawa Y, Suzuki H, Sakurai N, Aoki K, Saito K, Shibata D (2008). Cryopreservation and metabolic profiling analysis of *Arabidopsis* T87 suspension-cultured cells. CryoLetters.

[56] Towill L.E., Bonnart R, Volk G.M. (2006). Cryopreservation of *Arabidopsis thaliana* shoot tips. CryoLetters.

[57] Fuller B.J. (2004). Cryoprotectants: The essential antifreezes to protect life in the frozen state. CryoLetters.

[58] Sakai A, Kobayashi S, Oiyama I (1990). Cryopreservation of nucellar cells of navel orange (*Citrus sinensis* Osb. Var. *brasiliensis* Tanaka) by vitrification. Plant Cell Rep.

[59] Nishizawa S, Sakai A, Amano Y, Matsuzawa T (1993). Cryopreservation of asparagus (*Asparagus officinalis* L.) embryogenic suspension cells and subsequent plant-regeneration by vitrification. Plant Sci.

[60] Benson E.E. (2008). Cryopreservation of phytodiversity: A critical appraisal of theory and practice. Crit. Rev. Plant Sci.

[61] Volk G.M., Harris J.L., Rotindo K.E. (2006). Survival of mint shoot tips after exposure to cryoprotectant solution components. Cryobiology.

[62] Basu C (2008). Gene amplification from cryopreserved *Arabidopsis thaliana* shoot tips. Curr. Issues Mol. Biol.

[63] Agrawal A, Swennen R, Panis B (2004). A comparison of four methods for cryopreservation of meristems in banana (*Musa* spp.). CryoLetters.

[64] Panis B, Piette B, Swennen R (2005). Droplet vitrification of apical meristems: a cryopreservation protocol applicable to all Musaceae. Plant Sci.

[65] Carpentier S.C., Coemans B, Podevin N, Laukens K, Witters E, Matsumura H, Terauchi R, Swennen R, Panis B (2008). Functional genomics in an non-model crop: transcriptomics or proteomics?. Physiol. Plant.

[66] Carpentier S.C., Panis B, Vertommen A, Swennen R, Sergeant K, Renaut J, Laukens K, Witters E, Samyn B, Devreese B (2008). Proteome analysis of non-model plants: A challenging but powerful approach. Mass Spectrometry Rev.

[67] Samyn B, Sergeant K, Carpentier S, Debyser G, Panis B, Swennen R, Van Beeumen J (2007). Functional proteome analysis of the banana plant (*Musa* spp.) using de novo sequence analysis of derivatized peptides. J. Proteome Res.

[68] Carpentier S.C., Witters E, Laukens K, Van Onckelen H, Swennen R, Panis B (2007). Banana (*Musa* spp.) as a model to study the meristem proteome: Acclimation to osmotic stress. Proteomics.

[69] Zhu G.-Y., Guens J.M.C., Dussert S, Swennen R, Panis B (2006). Change in sugar, sterol and fatty acid composition in banana meristems caused by sucrose-induced acclimation and its effects on cryopreservation. Physiol. Plant.

[70] Peck L.S., Clark M.S., Clarke A, Cockell C.S., Convey P, Detrich H.W., Fraser K.P.P., Johnston I.A., Methe B.A., Murray A.E., Römisch K, Rogers A.D. (2005). Genomics: applications to Antarctic ecosystems. Polar Biol.

[71] Pura? J, Burns G, Thorne M.A.S., Grubor-Laj?i? G, Worland M.R., Clark M.S. (2008). Cold hardening processes in the Antarctic sprintail, *Cryptopygus antarcticus*: Clues from a microarray. J. Insect Physiol.

[72] Clark M.S., Worland M.R. (2008). How insects survive the cold: molecular mechanisms-a review. J. Comp. Physiol. B.

[73] Clark M.S., Thorne M.A.S., Pura? J, Grubor-Lajši? G, Kube M, Reinhardt R, Worland M.R. (2007). Surviving extreme polar winters by desiccation: clues from Arctic springtail (*Onychirus arcticus*) EST libraries. BMC Genomics.

[74] Storey K.B. (1999). Living in the cold: Freeze-induced gene responses in freeze-tolerant vertebrates. Clin. Experimental Pharmacology Physiol.

[75] Storey K.B. (2004). Strategies for exploration of freeze responsive gene expression: advances in vertebrate freeze tolerance. Cryobiology.

[76] Storey K.B. (2006). Reptile freeze tolerance: Metabolism and gene expression. Cryobiology.

[77] Margesin R, Neuner G, Storey K.B. (2007). Cold-loving microbes, plants, and animals-fundamental and applied aspects. Naturwissenschaften.

